# Preoperative prediction of Ki-67 and p53 status in meningioma using a multiparametric MRI-based clinical-radiomic model

**DOI:** 10.3389/fonc.2023.1138069

**Published:** 2023-05-23

**Authors:** Chung-Man Moon, Yun Young Lee, Doo-Young Kim, Woong Yoon, Byung Hyun Baek, Jae-Hyun Park, Suk-Hee Heo, Sang-Soo Shin, Seul Kee Kim

**Affiliations:** ^1^ Research Institute of Medical Sciences, Chonnam National University, Gwangju, Republic of Korea; ^2^ Department of Radiology, Chonnam National University Hospital, Gwangju, Republic of Korea; ^3^ Department of Artificial Intelligence Convergence, Chonnam National University, Gwangju, Republic of Korea; ^4^ Department of Radiology, Chonnam National University Medical School, Gwangju, Republic of Korea; ^5^ Department of Radiology, Chonnam National University Hwasun Hospital, Hwasun, Republic of Korea

**Keywords:** Ki-67, p53, meningioma, radiomics, machine learning

## Abstract

**Purpose:**

To investigate the utility of preoperative multiparametric magnetic resonance imaging (mpMRI)-based clinical-radiomic analysis combined with machine learning (ML) algorithms in predicting the expression of the Ki-67 proliferative index and p53 tumor suppressor protein in patients with meningioma.

**Methods:**

This multicenter retrospective study included 483 and 93 patients from two centers. The Ki-67 index was classified into high (Ki-67≥5%) and low (Ki-67<5%)-expressed groups, and the p53 index was classified into positive (p53≥5%) and negative (p53<5%)-expressed groups. Clinical and radiological features were analyzed using univariate and multivariate statistical analyses. Six ML models were performed with different types of classifiers to predict Ki-67 and p53 status.

**Results:**

In the multivariate analysis, larger tumor volumes (p<0.001), irregular tumor margin (p<0.001), and unclear tumor-brain interface (p<0.001) were independently associated with a high Ki-67 status, whereas the presence of both necrosis (p=0.003) and the dural tail sign (p=0.026) were independently associated with a positive p53 status. A relatively better performance was yielded from the model constructed by combined clinical and radiological features. The area under the curve (AUC) and accuracy of high Ki-67 were 0.820 and 0.867 in the internal test, and 0.666 and 0.773 in the external test, respectively. Regarding p53 positivity, the AUC and accuracy were 0.858 and 0.857 in the internal test, and 0.684 and 0.718 in the external test.

**Conclusion:**

The present study developed clinical-radiomic ML models to non-invasively predict Ki-67 and p53 expression in meningioma using mpMRI features, and provides a novel non-invasive strategy for assessing cell proliferation.

## Highlights

▪ Larger tumor volumes, irregular tumor margin, and unclear tumor-brain interface were independently associated with a high Ki-67 status.
▪ Presence of both necrosis and the dural tail sign were independently associated with a positive p53 status.
▪ A relatively better performance was yielded from the model constructed by combined clinical and radiological features.

## Introduction

Meningioma constitutes about one-third of primary central nervous system intracranial tumors with an annual incidence of about 5 per 100,000 individuals (1). More seriously, its incidence seems to rise constantly, and recurrence can occur causing significant morbidity and mortality (2). To date, it is difficult to evaluate and predict the biological behavior of meningioma. Therefore, mitotic and cell proliferation indices, tumor suppressor genes, angiogenesis intensity, inflammatory markers, histopathological results, and genetic and immunological levels should be considered in further risk assessments (3). However, because these variables are generally based on qualitative criteria, informational objectivity may be overshadowed (4). Therefore, the need for further quantitative criteria has emerged, triggering the quest for immunohistochemical markers of prognostic significance, which has been a routine practice in pathological diagnosis (5).

There are two quantitative and objective criteria used for the assessment of biological behavior as cell proliferative markers: Ki-67 and p53, which are most actively studied in meningioma for monitoring tumor aggressiveness, and objectively predicting tumor behavior in clinical intervention (4). The fact that the expression of Ki-67 and p53 can only be determined by using surgical or biopsy specimens from tumors remains a challenge in clinical practice. However, these are invasive procedures and may increase the risk of bleeding and the possibility of tumor metastasis (6, 7). Moreover, given that the evaluation of both markers relies on an expert pathologist’s decision, interobserver result variations are inevitable. Hence, it is essential to find an easy and non-invasive method for preoperatively assessing Ki67 and p53 expression to guide the surgical strategy decision and for prognostic prediction in meningioma.

Currently, as a quantitative method, radiomic analysis (RA) with machine learning (ML) algorithms, which can extract high-throughput computational features including tumor size, shape, texture patterns, and gray-level intensity from medical images, has attracted considerable interest in neurooncological research (8). Although recent studies (8, 9) identified ML-based RA using magnetic resonance imaging (MRI) datasets as a promising tool for grading meningioma, its ability to stratify the Ki-67 status in meningioma has been rarely studied (10, 11). Moreover, to our knowledge, the feasibility and value of the RA-based strategy on multiparametric MRI (mpMRI) for characterizing the p53 status in meningioma have not been validated.

In the present study, we set out to overcome the shortcomings of the manual assessment of Ki-67 and p53 and yet take advantage of the probable favorable role of both markers in the management of meningioma. We designed and suggested the use of ML-assisted methods with mpMR images for a more accurate prediction of tumor cells and Ki-67 and p53 expression, which may help prognosticate the heterogeneous clinical behavior, and reduce time-consuming and costly procedures.

Thus, the present study aimed to investigate the utility of preoperative mpMRI-based RA combined with ML algorithms in predicting the Ki-67 proliferative index and p53 tumor suppressor protein expression in patients with meningioma. Moreover, we developed ML classifiers trained with clinical-radiological features from qualitative MR imaging assessment, and further, the performances of these models were validated by an external dataset.

## Methods

### Patients

**Figure 1:**
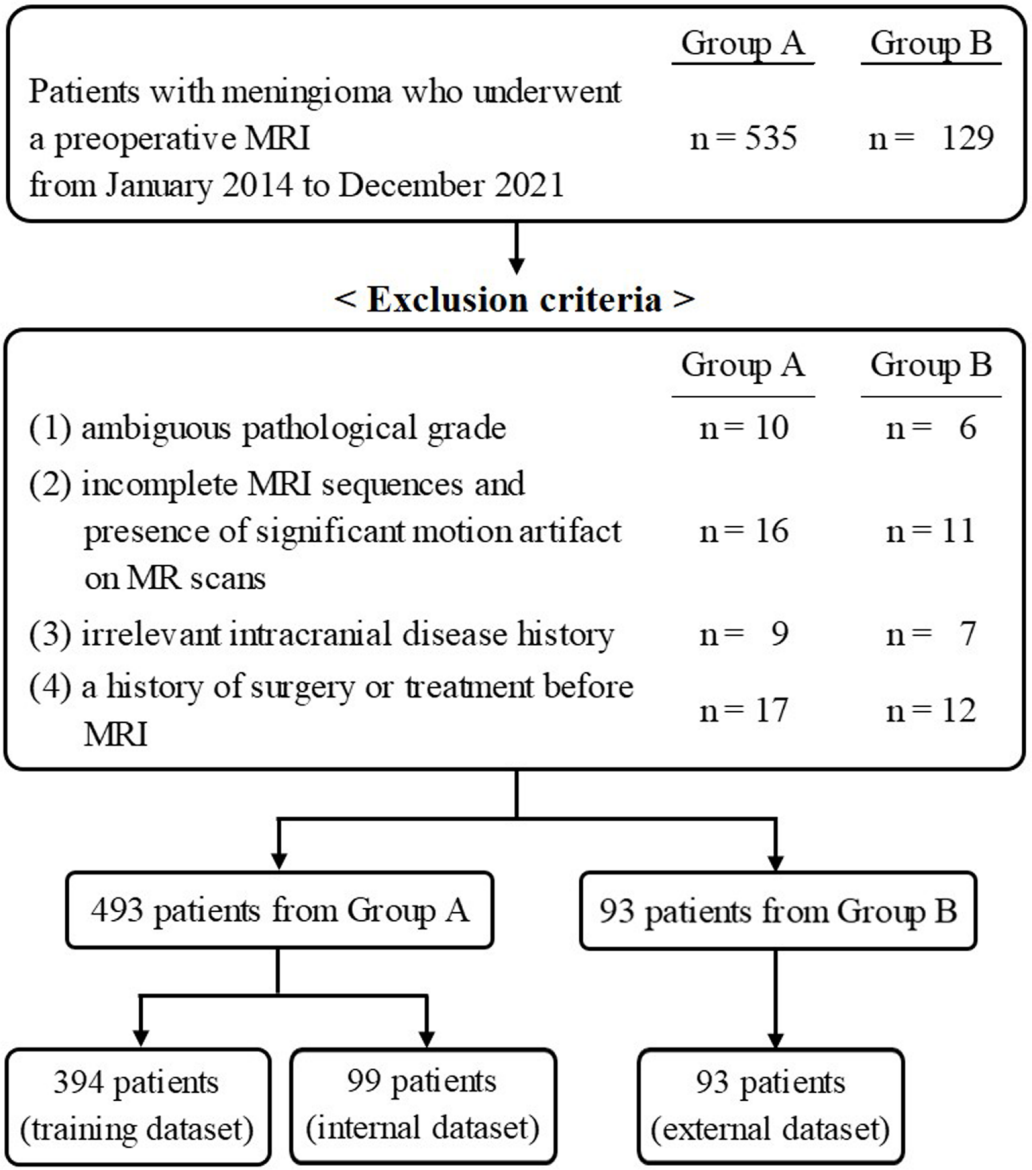
Flowchart of the study population including the inclusion and exclusion criteria.

This multicenter retrospective study was approved by the institutional review board of Chonnam National University Hospital, and was in conformation with the ethical guidelines of the 2008 Declaration of Helsinki. The requirement for written informed consent was waived due to the retrospective nature of the study. The patient selection flow chart is shown in **Figure 1**. From January 2014 to December 2021, 535 patients from Center A and 129 patients from Center B, who underwent a preoperative MRI, were initially recruited. The inclusion criteria were as follows: 1) histologically confirmed meningioma with a definite grade [according to the 2021 World Health Organization Classification of Tumors of the Central Nervous System (12)] and 2) available standard MR scans before any clinical intervention including biopsy and radiotherapy, consisting of T1- and T2-weighted images (T1WI, T2WI), T1-contrast enhanced (T1-CE), fluid-attenuated inversion recovery (FLAIR), and apparent diffusion coefficient (ADC) data. The exclusion criteria were as follows: 1) ambiguous pathological grade (n = 16); 2) incomplete MRI sequences and the presence of significant motion artifacts on MR scans (n = 27); 3) irrelevant intracranial disease history (n = 16); and 4) a history of surgery or treatment before MRI (n = 29). Finally, 483 patients and 93 patients from Center A and Center B, respectively, were retained.

### Immunohistochemistry

Histopathologic data were determined based on the surgically removed tissue. In all patients, the Ki-67 labeling index and p53 expression were assessed by immunohistochemistry (IHC) and quantified by a pathologist. Although these expressions were determined as a prognostic predictor for patients with meningioma, the optimal threshold value had not yet been identified. Previous studies (10, 11, 13) defined a cut-off point for high Ki-67 expression at ≥ 5% positive cells, demonstrating that the recurrence rate of meningioma was higher in patients with a Ki-67 labeling index of 5% or more. The samples in the present study were classified into high Ki-67 expression (Ki-67 ≥ 5%) and low Ki-67 expression (Ki-67 < 5%). For p53 status, wild-type or nuclei with positive staining of < 5% was considered negative, whereas an abnormal complete absence or nuclei with positive staining of ≥ 5% considered as positive (14, 15), which was based on previous reports that the progression-free survival decreased remarkably at a p53-positive rate of 5% (16), and expressed p53 protein of ≥ 5% was frequently observed in recurrent meningioma with malignant transformation (17).

### MRI protocols

Preoperative MRI studies were performed at the two centers. In center A, all MR imaging examinations were performed on 3T scanners (Magnetom TimTrio, Skyra, Vida; Siemens Healthcare). They used similar imaging protocols, which included the following sequences: T1WI (TR/TE = 2400 − 2540 ms/9.4 ms; matrix = 384 × 269), T2WI (TR/TE = 3500 − 3700 ms/100 − 105 ms; matrix = 448 × 311), FLAIR (TR/TE 7000 ms/80 − 96 ms; matrix = 384 × 230), and T1-CE (TR/TE 149 − 164 ms/3 − 4.4 ms; matrix = 480 × 381). An FOV of 230 mm × 230 mm, slice thickness of 4 mm, and no gap were applied in all images. The contrast-enhanced MR scans were acquired after a bolus injection of 0.2 mL/kg of contrast agent. Diffusion-weighted imaging (DWI) was acquired with the following parameters: TR/TE = 5200 − 5500 ms/72 − 80 ms, matrix = 128 − 160 × 128 − 160, FOV = 230 mm × 230 mm, slice thickness = 4 mm, no slice gap, and two b values (b = 0 and 1000 s/mm^2^). ADC maps were automatically generated on the MR system.

In Center B, mpMRI was performed using 3T MR scanners (MAGNETOM TimTrio, Vida; Siemens Healthcare, Discovery 750; GE Healthcare, Ingenia CX: Philips Healthcare). The detailed protocols included the following sequences: T1WI (TR/TE = 2000 − 2400 ms/10 − 13 ms; matrix = 320 − 256 × 230 − 287), T2WI (TR/TE = 3000 − 6000 ms/80 − 100 ms; matrix = 400 − 512 × 259 − 400), FLAIR (TR/TE 4800 − 9400 ms/88 − 340 ms; matrix = 256 − 384 × 204 − 264), and T1-CE (TR/TE 287 − 350 ms/2.5 − 4.6 ms; matrix = 320 − 400 × 224 − 321). An FOV of 230 − 240 mm × 230 − 240 mm, slice thickness of 5 mm, and a gap of 0.5 mm were applied in all images. T1-CE images were acquired after a bolus injection of 0.2 mL/kg of contrast agent. DWI was acquired with the following parameters: TR/TE = 4300 − 7600 ms/54 − 78.9 ms, matrix = 120 − 160 × 120 − 160, FOV = 240 mm × 240 mm, slice thickness = 4 mm, no slice gap, and two b values (b = 0 and 1000 s/mm^2^). ADC maps were automatically generated on the MR system.

### Radiological evaluation of MRI data

Two neuroradiologists with 8 and 13 years of experience in brain MR imaging, who were blinded to the pathological results, reviewed the MR images. They evaluated the radiological characteristics of the meningioma as a mostly qualitative interpretation with regard to tumor volume (mm^3^), edema volume (mm^3^), edema/tumor volume ratio, ADC value (×10^-3^ mm^2^/s), internal enhancement characteristics of the main tumor (homogeneous or heterogeneous), necrosis and the dural tail sign (presence or absence), tumor margin (regular or irregular), and tumor-brain interface (clear or unclear).

### Image preprocessing and tumor segmentation

**Figure 2:**
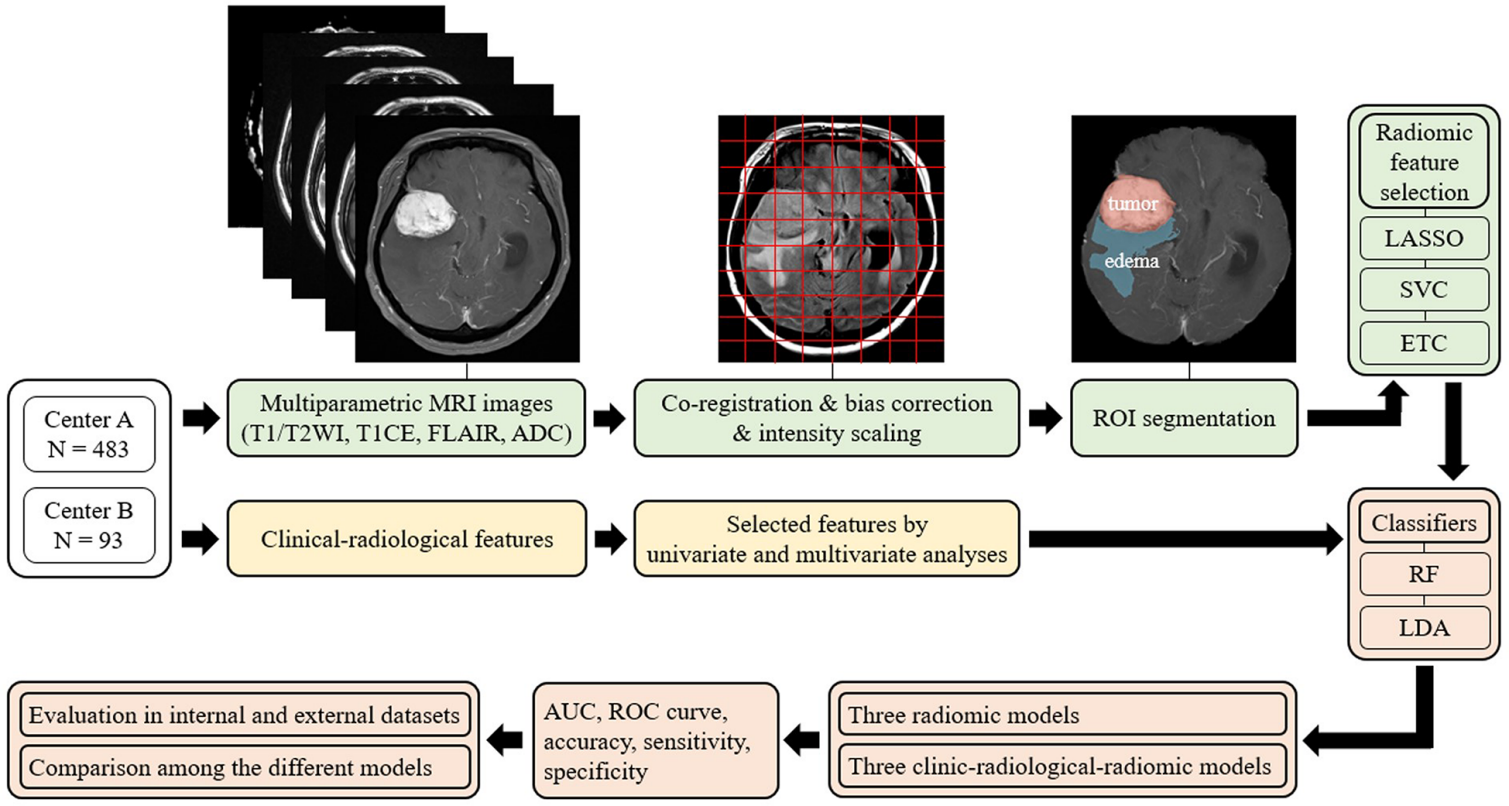
Overview of the radiomic analysis framework used to develop a machine-learning model to predict Ki-67 and p53 expression in patients with meningioma. LASSO, least absolute shrinkage and selection operator; SVC, support vector classification; ETC, extra tree classification; RF, random forest; LDA, linear discriminant analysis; AUC, areas under the curve; ROC curve, receiver-operating characteristic curve.

A schematic showing the process of image processing and ML analysis is shown in **Figure 2**. Image preprocessing was required to standardize radiomic feature extraction. Before analysis, N4 bias correction was applied to remove low-frequency intensity nonuniformity from the T1-CE, T1WI, T2WI, and FLAIR images using Advanced Normalization Tools (18). Subsequently, image preprocessing for each of the patients included coregistration of all MRI sequences upon its corresponding axial thin-cut (0.5 mm) T1-CE sequence, and resampling of the images to 1 × 1 × 1 mm^3^ resolution. Subsequently, to minimize inherent differences in pixel intensities across three different MR scanners, the gray-level intensity for all image volumes was scaled in the range of 0 − 255 after removing pixels with outlier values (19).

Among all MRIs, contrast-enhanced images most clearly describe the tumor boundary and were selected for radiomic feature extraction. The entire tumor volumes were segmented to create the volume of interest (VOI) of T1-CE images with a semiautomatic method based on a signal intensity threshold and edge-based algorithms. Peritumoral edema was then identified on the FLAIR sequence, using the 3D slicer software (version 4.11, Boston, MA, USA) to satisfy image segmentation by the neuroradiologists blinded to patients’ clinical information with Ki-67 and p53 levels (18).

### Radiomic feature extraction

The radiomic features were retrieved using an open-source python package, PyRadiomics v2.2.0. In total, 1605 radiomic features were initially retrieved, including three-dimensional shape features (n = 14) from enhancing tumor and peritumoral edema regions, first-order radiomic features (n = 18), and higher-order radiomic features from four different matrices, including gray-level co-occurrence matrix (GLCM) (n = 24), gray-level size zone matrix (GLSZM) (n = 16), gray-level run length matrix (GLRLM) (n = 16), neighboring grey tone difference matrix (NGTDM) (n = 5), and gray-level dependence matrix (GLDM) (n = 14). Next, the radiomic features were standardized by removing the mean and scaling to unit variance.

Regarding feature reproducibility, 30 images from randomly selected patients were chosen to evaluate the reproducibility of the radiomic features. The tumor segmentation and feature extraction were performed by two neuroradiologists twice at one-week intervals. The intraclass correlation coefficient (ICC) was used to assess the interobserver reproducibility of radiomic features based on a two-way mixed effects model and ICC values ≥ 0.70 or 0.90 was considered good or excellent reproducibility for subsequent investigation.

### Feature selection and classifier model training for radiomic analysis

The extensive number of extracted texture features must be selected properly at first to avoid overfitting the ML algorithms. For clinical features and radiological features, multivariate logistic regression was performed to select the significantly correlated features for the ML model, and p-values < 0.05 were considered statistically significant in the multivariate analysis. Moreover, for radiomic features, three methods were independently used to select relatively important features, including least absolute shrinkage and selection operator (LASSO), support vector classification (LinearSVC), and extra tree classification (ETC) (11). The misclassification error was determined by a tuning parameter (Lambda). As the Lambda gets smaller, some coefficients may be shrunk towards zero (20) (**Supplementary Figure 1**). We then selected the Lambda for which the cross-validation error was the smallest.

Three radiomic-based ML models and three combined clinical-radiologic and radiomic-based ML models were established for a five-fold cross-validation to predict the Ki-67 and p53 expression in patients with meningioma. The patients from Center A were randomly split into training and independent test sets at a ratio of 4:1; patients from Center B were used as the external test group. Random forest (RF) and linear discriminant analysis (LDA) were used as ML classifiers. Furthermore, considering that the performance of the classifier would be influenced by the imbalanced sample ratio between low-grade and high-grade groups for Ki-67, and negative and positive groups for p53, the Adaptive Synthetic Sampling Approach for Imbalanced Learning with default parameter was performed to balance the sample numbers (21). To evaluate the performance of radiomics-based predictive models for Ki-67 and p53 expression, area under the curve (AUC), receiver operating characteristic curve (ROC curve), accuracy, sensitivity, and specificity were calculated for both the test and validation sets using Python programming language (version 3.9).

### Statistical analysis

Percentages and frequencies were used for categorical variables, and means and standard deviation were used for continuous variables. Univariate analysis was conducted to select the significant clinical and radiological characteristics in Ki-67 low-and high-expression groups, and p53 positive and negative groups in the training cohort. Multivariate analysis was then performed to construct a clinical-radiological model. A p-value < 0.05 was considered to represent statistical significance. Interobserver agreement was evaluated by calculating the ICCs of the extracted features, and only the radiomic features with high ICCs (ICCs ≥ 0.75) were taken into modeling. Statistical analysis was performed with IBM SPSS statistics for windows version 22 (SPSS Inc, Chicago, IL).

## Results

### Clinical characteristics

The clinical characteristics and demographics of the 576 patients are summarized in **Table 1**. The mean patient age was 59.89 ± 13.05 years (range: 22 − 88), and the sex ratio of the study cohort was Male: Female =167: 409. For the 483 cases from Center A, the mean Ki-67 level was 4.41 ± 6.95% (range: 1 − 60), consisting of 299 patients with Ki-67 < 5% and 184 patients with Ki-67 ≥ 5%. For the 93 cases from Center B, the mean Ki-67 level was 3.13 ± 4.07% (range: 1 − 20), consisting of 68 patients with Ki-67 < 5% and 25 patients with Ki-67 ≥ 5%. In addition, the proportion of patients with a p53-positive status was 242 cases (50.10%) in Center A and 54 cases (58.06%) in Center B.

**Table 1:** Clinical and radiological characteristics of the patients.

Characteristics	Center A	Center B
	(n = 483)	(n = 93)
Mean age (years)	59.44 ± 13.34	59.79 ± 13.17
Men	149 (30.85%)	18 (19.35%)
Tumor volume (cm^3^)	35.99 ± 35.78	27.76 ± 25.24
Edema volume (cm^3^)	36.59 ± 52.92	24.82 ± 42.13
Edema/tumor volume ratio	1.38 ± 3.45	1.30 ± 3.47
ADC value (×10^-3^ mm^2^/s)	0.80 ± 0.20	0.79 ± 0.23
Enhancement		
Homogeneous	282 (58.39%)	60 (64.52%)
Heterogeneous	201 (41.61%)	33 (35.48%)
Presence of necrosis	104 (21.53%)	16 (17.20%)
Presence of dural tail	385 (79.71%)	79 (84.95%)
Tumor margin		
Regular	276 (57.14%)	71 (76.34%)
Irregular	207 (42.86%)	22 (23.66%)
Tumor-brain interface		
Clear	393 (81.37%)	82 (88.17%)
Unclear	90 (18.63%)	11 (11.83%)
Ki-67 ≥ 5%	184 (38.10%)	25 (26.88%)
Ki-67 < 5%	299 (61.90%)	68 (73.12%)
p53 positive	242 (50.10%)	54 (58.06%)
p53 negative	241 (49.90%)	39 (41.94%)

### Clinical and radiological features related to the Ki-67 and p53 indexes

The results of the univariate analysis indicated that larger tumor volumes (p < 0.001), the presence of the dural tail sign (p = 0.01), irregular tumor margin (p = 0.03), and unclear tumor-brain interface (p < 0.001) were significantly associated with a high Ki-67 expression. In the multivariate analysis that was performed using the variables showing significant p-value in the univariate analysis, larger tumor volumes (p < 0.001), irregular tumor margin (p < 0.001), and unclear tumor-brain interface (p < 0.001) were independently associated with a high Ki-67 expression.

In the univariate analysis, the presence of necrosis (p = 0.001), the dural tail sign (p = 0.04), and an irregular tumor margin (p = 0.03) were significantly associated with a positive p53 status. Multivariate analysis revealed that the presence of both necrosis (p < 0.001) and the dural tail sign (p = 0.03) were independently associated with a positive p53 status. All outcomes from the univariate and multivariate analyses are demonstrated in **Table 2**.

**Table 2:** Univariate and multivariate analyses for predicting Ki-67 and p53 expression.

	Univariate analysis		Multivariate analysis	
Parameters	β (95% CI)	p-value	β (95% CI)	p-value
*Ki-67*				
Age (years)	0.00 (0.00 − 0.00)	0.82	–	–
Sex	0.03 (-0.05 − 0.11)	0.43	–	–
Tumor volume (cm^3^)	0.00 (0.00 − 0.00)	**0.00**	17.74 (12.06 − 23.42)	**0**
Edema volume (cm^3^)	0.00 (0.00 − 0.00)	0.93	–	–
Edema/tumor volume ratio	0.00 (-0.01 − 0.02)	0.77	–	–
ADC value (×10^-3^ mm^2^/s)	-0.10 (-0.29 − 0.09)	0.28	–	–
Enhancement	-0.05 (-0.14 − 0.04)	0.28	–	–
Necrosis	0.08 (-0.03 − 0.18)	0.15	–	–
Dural tail	0.13 (0.03 − 0.23)	**0.01**	0.06 (0.00 − 0.13)	0.07
Tumor margin	0.11 (0.01 − 0.20)	**0.03**	0.24 (0.16 − 0.32)	**0**
Tumor-brain interface	0.18 (0.06 − 0.31)	**0.00**	0.18 (0.11 − 0.24)	**0**
p53				
Age (years)	0.00 (0.00 − 0.00)	0.66	–	–
Sex	0.06 (-0.03 − 0.14)	0.18	–	–
Tumor volume (cm^3^)	0.00 (0.00 − 0.00)	0.33	–	–
Edema volume (cm^3^)	0.00 (0.00 − 0.00)	0.11	–	–
Edema/tumor volume ratio	0.00 (-0.01 − 0.02)	0.7	–	–
ADC value (×10^-3^ mm^2^/s)	0.02 (-0.22 − 0.19)	0.88	–	–
Necrosis	0.18 (0.07 − 0.30)	**0.00**	0.10 (0.03 − 0.17)	**0**
Dural tail	0.11 (0.01 − 0.22)	**0.04**	0.07 (0.01 − 0.14)	**0.03**
Tumor margin	0.12 (0.01 − 0.22)	**0.03**	0.06 (-0.02 − 0.14)	0.14
Tumor-brain interface	-0.06 (-0.19 − 0.08)	0.41	–	–

Bolded values are significant at p < 0.05.

### Radiomic feature selection

Based on the results of feature selection, 20 radiomic features were determined to be important and were separately introduced into predictive models wrapped by the RF and LDA algorithms. The distribution of each selected feature is demonstrated in **Table 3**. Three radiomic-based models were constructed based on radiomic features, and three combined clinical-radiologic and radiomic models were constructed using different combinations of radiomic features and clinical features.

**Table 3:** The numbers of selected features *via* different approaches.

	LASSO		SVC		ETC		
	Feature No.	Feature categories	Feature No.	Feature categories	Feature No.	Feature categories	
*Ki-67*							
T1-weighted	5	First-order (n = 2), GLRLM (n = 1), GLDM (n = 2)	4	First-order (n = 3), GLDM (n = 1)	4	First-order (n = 4)	
T1-CE	7	First-order (n = 4), GLSZM (n = 2), GLRLM (n = 1)	8	First-order (n = 3), GLCM (n = 2), GLSZM (n = 2), GLDM (n = 1)	7	First-order (n = 5), GLSZM (n = 1), Shape (n = 1)	
T2-weighted	5	First-order (n = 3), GLSZM (n = 1), GLRLM (n = 1)	4	First-order (n = 3), GLRLM (n = 1)	6	First-order (n = 4), GLCM (n = 1), GLSZM (n = 1)	
FLAIR	−	−	1	First-order (n = 1)	−	−	
ADC	3	First-order (n = 2), GLSZM (n = 1)	3	First-order (n = 1), GLRLM (n = 2)	3	First-order (n = 3)	
*p53*							
T1-weighted	4	First-order (n = 2), GLRLM (n = 1), GLDM (n = 1)	5	First-order (n = 2), GLRLM (n = 2), GLDM (n = 1)	4	GLSZM (n = 2), NGTDM (n = 2)	
T1-CE	6	First-order (n = 1), GLSZM (n = 2), NGTDM (n = 3)	7	GLCM (n = 1), NGTDM (n = 4), GLDM (n = 2)	7	First-order (n = 1), GLCM (n = 2), NGTDM (n = 4)	
T2-weighted	3	First-order (n = 1), GLSZM (n = 2)	1	First-order (n = 1)	1	First-order (n = 1)	
FLAIR	4	First-order (n = 2), GLRLM (n = 1), GLDM (n = 1)	4	First-order (n = 1), GLSZM (n = 2), GLDM (n = 1)	4	First-order (n = 1), GLCM (n = 1), GLSZM (n = 2)	
ADC	3	First-order (n = 2), GLSZM (n = 1)	3	First-order (n = 2), NGTDM (n = 1)	4	First-order (n = 1), GLCM (n = 2), GLSZM (n = 1)	

### The diagnostic performance of the prediction models

Among the radiomic-based predictive models for Ki-67 and p53 expression, a relatively better performance was yielded from the model constructed by the features selected by LASSO and classified by RF. The AUC, accuracy, sensitivity, and specificity of high Ki-67 were 0.756, 0.813, 0.576, and 0.936 in the internal test, respectively, and the model showed a decline in these indexes in the external test (external AUC: 0.677, accuracy: 0.751, sensitivity: 0.516, and specificity: 0.837). Concerning the positive p53 status, the AUC, accuracy, sensitivity, and specificity were 0.729, 0.731, 0.743, and 0.715 in the internal test, respectively. These indexes were 0.614, 0.631, 0.506, and 0.722, respectively, in the external test.

**Figure 3:**
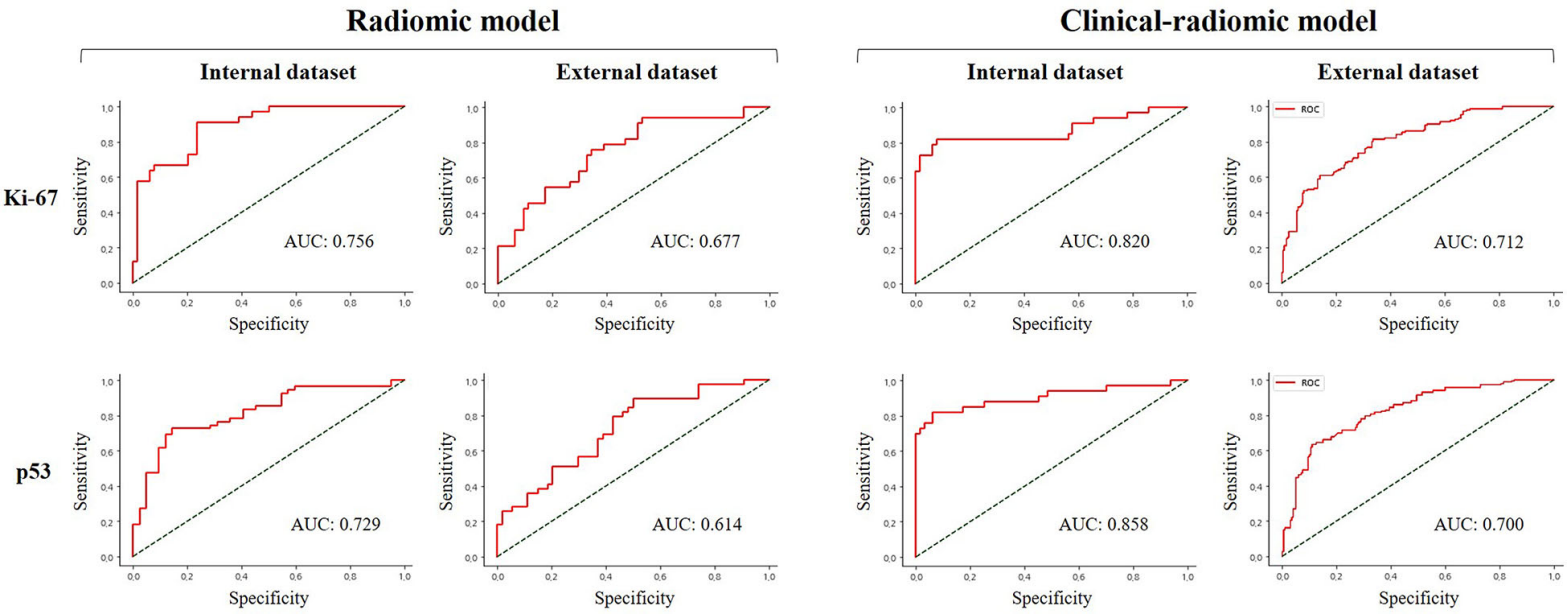
Comparison of receiver operating characteristic curve for prediction of Ki-67 and p53 expression in the internal and external validation dataset by the different models with LASSO and RF performance.

When clinical and radiological features were combined, this approach through LASSO and RF showed improvement and achieved the highest performance among all the models for predicting a high Ki-67 expression. This was with an AUC of 0.820, accuracy of 0.867, sensitivity of 0.671, and specificity of 0.969 in the internal test, and an AUC of 0.666, accuracy of 0.773, sensitivity of 0.435, and specificity of 0.896 in the external test. Regarding the prediction of positive p53 status, the AUC, accuracy, sensitivity, and specificity were 0.858, 0.857, 0.877, and 0.894 in the internal test, and 0.684, 0.718, 0.462, and 0.905 in the external test. The ROC curves of the LASSO and RF models are illustrated in **Figure 3**. All model performances are demonstrated in **Table 4** for Ki-67 and **Table 5** for p53.

**Table 4:** Predictive model performance for Ki-67 in the internal and external test.

Models	Features/classification	Test	Accuracy	95% CI	AUC	95% CI	Sensitivity	95% CI	Specificity	95% CI
Radiomics	Lasso+RandomForest	Internal test	0.813	0.73 - 0.89	0.756	0.666 - 0.845	0.576	0.407 - 0.75	0.936	0.876 - 0.986
		External test	0.751	0.669 - 0.83	0.677	0.567 - 0.778	0.516	0.322 - 0.708	0.837	0.753 - 0.917
	Lasso+LDA	Internal test	0.813	0.74 - 0.88	0.756	0.669 - 0.841	0.576	0.405 - 0.741	0.937	0.873 - 0.985
		External test	0.753	0.66 - 0.83	0.679	0.582 - 0.779	0.518	0.344 - 0.708	0.839	0.753 -0.921
	LinearSVC+RandomForest	Internal test	0.796	0.71 - 0.87	0.721	0.634 - 0.81	0.487	0.31 - 0.656	0.955	0.901 - 1
		External test	0.751	0.67 - 0.83	0.615	0.526 - 0.719	0.317	0.142 - 0.5	0.913	0.845 - 0.972
	LinearSVC+LDA	Internal test	0.795	0.71 - 0.87	0.72	0.636 - 0.811	0.486	0.323 - 0.648	0.954	0.898 - 1
		External test	0.753	0.67 - 0.83	0.616	0.526 - 0.708	0.321	0.156 - 0.5	0.91	0.845 - 0.972
	ExtraTree+RandomForest	Internal test	0.773	0.689 - 0.85	0.733	0.636 - 0.821	0.608	0.448 - 0.769	0.857	0.765 - 0.94
		External test	0.783	0.7 - 0.86	0.698	0.603 - 0.796	0.515	0.333 - 0.694	0.882	0.8 - 0.946
	ExtraTree+LDA	Internal test	0.771	0.69 - 0.85	0.729	0.635 - 0.822	0.6	0.428 - 0.766	0.859	0.77 - 0.937
		External test	0.785	0.7 - 0.87	0.7	0.598 - 0.811	0.518	0.32 - 0.718	0.882	0.807 - 0.951
Clinics+ Radiomics	Lasso+RandomForest	Internal test	0.867	0.81 - 0.93	0.82	0.741 - 0.897	0.671	0.514 - 0.821	0.969	0.923 - 1
		External test	0.743	0.66 - 0.82	0.712	0.611 - 0.808	0.643	0.45 -0.809	0.78	0.684 - 0.875
	Lasso+LDA	Internal test	0.866	0.79 - 0.93	0.817	0.727 - 0.896	0.665	0.499 - 0.818	0.968	0.919 - 1
		External test	0.777	0.69 - 0.85	0.67	0.569 - 0.771	0.441	0.25 - 0.631	0.899	0.823 - 0.96
	LinearSVC+RandomForest	Internal test	0.846	0.77 - 0.91	0.796	0.705 - 0.88	0.639	0.47 - 0.8	0.953	0.893 - 1
		External test	0.732	0.64 - 0.82	0.628	0.534 - 0.74	0.403	0.217 - 0.613	0.854	0.774 - 0.925
	LinearSVC+LDA	Internal test	0.847	0.77 - 0.91	0.796	0.705 - 0.881	0.639	0.468 - 0.794	0.953	0.892 - 1
		External test	0.731	0.64 - 0.81	0.626	0.516 - 0.725	0.398	0.206 - 0.577	0.854	0.767 - 0.931
	ExtraTree+RandomForest	Internal test	0.815	0.74 - 0.89	0.757	0.67 - 0.843	0.576	0.407 - 0.743	0.938	0.876 - 0.985
		External test	0.773	0.68 - 0.85	0.666	0.562 - 0.767	0.435	0.25 - 0.629	0.896	0.823 - 0.96
	ExtraTree+LDA	Internal test	0.815	0.74 - 0.89	0.758	0.663 - 0.838	0.578	0.4 - 0.736	0.937	0.873 - 0.985
		External test	0.744	0.66 - 0.83	0.713	0.614 - 0.82	0.644	0.444 - 0.826	0.781	0.684 - 0.873

**Table 5:** Predictive model performance for p53 in the internal and external test.

Models	Features/classification	Test	Accuracy	95% CI	AUC	95% CI	Sensitivity	95% CI	Specificity	95% CI
Radiomics	Lasso+RandomForest	Internal test	0.731	0.649 - 0.81	0.729	0.642 0.815	0.743	0.618 - 0.84	0.715	0.585 - 0.842
		External test	0.631	0.54 - 0.73	0.614	0.516 - 0.71	0.506	0.355 - 0.666	0.722	0.599 - 0.828
	Lasso+LDA	Internal test	0.731	0.65 - 0.82	0.728	0.644 - 0.811	0.744	0.627 - 0.854	0.713	0.584 - 0.837
		External test	0.636	0.55 - 0.74	0.619	0.527 - 0.717	0.516	0.357 - 0.681	0.721	0.599 - 0.839
	LinearSVC+RandomForest	Internal test	0.71	0.62 - 0.8	0.719	0.635 - 0.803	0.653	0.534 - 0.769	0.786	0.666 - 0.895
		External test	0.657	0.57 - 0.75	0.637	0.547 - 0.726	0.515	0.365 - 0.657	0.759	0.655 - 0.866
	LinearSVC+LDA	Internal test	0.71	0.62 - 0.8	0.719	0.63 - 0.806	0.654	0.523 - 0.775	0.783	0.653 - 0.894
		External test	0.657	0.56 - 0.74	0.636	0.542 - 0.733	0.509	0.361 - 0.666	0.763	0.655 - 0.866
	ExtraTree+RandomForest	Internal test	0.659	0.56 - 0.75	0.656	0.559 - 0.751	0.674	0.546 - 0.792	0.639	0.499 - 0.772
		External test	0.733	0.64 - 0.82	0.692	0.611 - 0.777	0.437	0.292 - 0.589	0.946	0.88 - 1
	ExtraTree+LDA	Internal test	0.657	0.56 - 0.75	0.655	0.556 - 0.743	0.672	0.538 - 0.793	0.638	0.477 - 0.777
		External test	0.73	0.64 - 0.82	0.69	0.612 - 0.773	0.435	0.285 - 0.593	0.944	0.883 - 1
Clinics+ Radiomics	Lasso+RandomForest	Internal test	0.857	0.79 - 0.92	0.858	0.786 - 0.921	0.877	0.759 - 0.944	0.894	0.755 - 0.95
		External test	0.731	0.64 - 0.81	0.7	0.615 - 0.767	0.436	0.294 - 0.581	0.944	0.872 - 1
	Lasso+LDA	Internal test	0.854	0.789 - 0.92	0.856	0.782 - 0.924	0.851	0.758 - 0.947	0.855	0.736 - 0.954
		External test	0.719	0.64 - 0.8	0.683	0.603 - 0.767	0.458	0.314 - 0.6	0.908	0.823 - 0.967
	LinearSVC+RandomForest	Internal test	0.794	0.71 - 0.87	0.793	0.704 - 0.869	0.8	0.689 - 0.896	0.787	0.644 - 0.902
		External test	0.71	0.62 - 0.8	0.683	0.593 - 0.767	0.516	0.355 - 0.666	0.85	0.75 - 0.938
	LinearSVC+LDA	Internal test	0.793	0.72 - 0.88	0.792	0.712 - 0.871	0.8	0.685 - 0.91	0.784	0.655 - 0.9
		External test	0.707	0.619 - 0.8	0.678	0.585 - 0.77	0.506	0.349 - 0.659	0.85	0.759 - 0.933
	ExtraTree+RandomForest	Internal test	0.782	0.7 - 0.87	0.782	0.693 - 0.869	0.779	0.666 - 0.883	0.784	0.651 - 0.913
		External test	0.718	0.63 - 0.8	0.684	0.601 - 0.763	0.462	0.319 - 0.609	0.905	0.83 - 0.98
	ExtraTree+LDA	Internal test	0.786	0.7 - 0.87	0.785	0.7 - 0.871	0.784	0.677 - 0.889	0.788	0.659 - 0.9
		External test	0.73	0.65 - 0.81	0.689	0.609 - 0.767	0.433	0.289 - 0.575	0.945	0.875 - 1

## Discussion

ML using radiomic features derived from preoperative MR images may provide prognostic insights to predict the biological behavior of meningioma. To our knowledge, this is a novel study that establishes a radiomic classifier using mpMRI images to predict both Ki-67 and p53 expression. In the present study, we trained an ML MRI radiomics-based model that can be used to assess the predictive efficiency of a Ki-67 proliferation index of ≥ 5% and p53 positive expression and to help guide surgical timing and the choice of operative strategy. Ki-67 and p53-associated features were each screened by feature selection approaches including the LASSO, LinearSVC, and ETC. Then, different ML models of RF and LDA could predict Ki-67 and p53 expression. On the other hand, it is important to be aware of the problem of overfitting that occurs when the learning algorithm describes the random error or noise instead of the underlying data relationship. Consequently, in this study, the Bootstrap method and cross-validated prediction were applied to strengthen the robustness of the obtained data. Subsequently, an ML-based prediction model incorporating mpMRI features showed good diagnostic performance for predicting Ki-67 and p53 expression with an average AUC of 0.736 and 0.701, respectively, from three radiomic-based ML models in the internal dataset.

Furthermore, our ML classifier was built using both clinical-radiological variables and tumoral radiomic features, and the resultant integrated clinical-radiomic models achieved validation for classifying a Ki-67 of ≥ 5% and identifying p53 positivity with an average AUC of 0.791 and 0.811 from all ML models in the internal dataset, respectively. The clinical-radiomic model outperformed, indicating the potential use of radiomics in predicting Ki-67 and p53 expression. Overall, our results indicate that the Ki-67 and p53 status can be predicted using non-invasive radiological data, and that an ML approach that integrates multivariate features is more effective and robust than individual features (22). Hypothetically, we supposed that the performance in the test set should be slightly attenuated because it seems unlikely that whatever model that performs best on the training set would perform equally well on every other unseen data set. Further studies with the inclusion of more data are required to strengthen these findings.

Similar to our findings, few studies (10, 11) applied radiomic-based ML to predict the Ki-67 expression in meningioma. The models involving multiparametric feature sets from multiple MR sequences, including T1WI, T2WI, T1-CE, and FLAIR were superior to models involving single-sequence feature sets (23). Another recent study (11) demonstrated that the clinical-radiologic model outperformed the single radiomic model, showing an AUC of 0.810 and 0.557 from the LASSO and LDA models in the internal test and external test, respectively. On the other hand, a study that used a single radiomic model reported that p53 status in patients with glioma can be quantitatively predicted through 86 radiomic features from preoperative MRI, with an accuracy of 65.2% (AUC = 71.9%) (24). As the first study on the p53 status in meningioma, when constructing the clinical-radiologic model with mpMRI in our study, the accuracy was 0.854 (AUC = 0.856) compared to that of our single radiomic model (accuracy: 0.731; AUC = 0.728).

Through various ML algorithms applied to RA, we finally extracted two sets of 20 Ki-67- and p53-related features from each patient, which consisted of the first-order features adding more advanced high-order features. The entropy belongs to the GLCM feature pool reflecting the intensity of the spatial distribution, which means that the larger entropy value represents a greater tumor heterogeneity (25). Regarding the uniformity of the tumor texture of the GLSZM, high-grade meningiomas are featured by a larger proportion of tissue disruption, and thus a higher heterogeneity of the distribution of cells in the tumor lesions compared with low-grade lesions (25). The non-uniformity of the GLRLM is another important radiomic feature, which is sensitive in reflecting the heterogeneity within the contoured area, such as positive capsular enhancement, an indistinguishable tumoral border, and heterogeneous tumor enhancement (26). The NGTDM features, including busyness, contrast, and coarseness, may reflect microscopic heterogeneity within the tumors (27). The heterogeneous distribution of cell density was quantified by these features in our study. Dependency uniformity features of the GLDM were found to be significantly effective in predicting the Ki-67 proliferation index in meningioma (11). These radiomic features can be used to reflect the spatial heterogeneity of meningioma of different pathological grades for Ki-67 and p53.

In particular, our study revealed that the radiomic features derived from T1-CE images contributed the highest number of selected features in both Ki-67 and p53, which were useful for predicting immunohistochemical markers of meningioma. It has been reported that most meningiomas exhibit a marked enhancement on T1-CE images because of abundant blood supply in which the tumor boundaries can be clearly exhibited. Moreover, when comparing the diagnostic performance among T1WI, T2WI, and T1-CE sequences, a predictive model to obtain more robust results using presurgical T1-CE data showed a satisfactory capability to differentiate the pathological grade of meningioma (18, 28). Based on these findings, we successfully developed an ML model based on T1-CE images for predicting Ki-67 and p53 expression.

After multivariate analysis of clinical features with the expression of Ki-67 and p53, we found that there were significant correlations−consistent with the findings of other studies (29, 30). Regarding the association of tumor necrosis with high Ki-67 expression, it could be assumed that a high proliferation of Ki-67 may exhaust the oxygen supply of their vascular system, resulting in prolonged hypoxia and subsequent necrosis with decreased cellularity (31). Thus, preoperatively, a larger tumor volume, irregular tumor margin, and unclear tumor-brain interface, and the presence of necrosis and the dural tail sign may potentially influence the histological status of the Ki-67 and p53 indexes, representing the development and growth of meningioma.

Our primary experiments have demonstrated the clinical feasibility of predicting tumor immunohistochemical markers based on mpMRI images of patients with meningioma, and provide further information regarding tumor proliferation and relevant biological behavior before any invasive examinations. Moreover, in the different types of ML classifiers successfully conducted in this study, different classifiers demonstrated different predictive performances, implying that the choice of the classifier model type had an important influence on the outcomes. In clinical practice, the approach for predicting the Ki-67 and p53 indexes may provide guidance regarding the imaging surveillance and decision-making regarding surgical intervention. When expecting the increased risk of meningioma development, the patients diagnosed who elevated Ki-67 and p53 could be counseled to earlier undergo surgery to maximize the extent of tumor resection with minimizing the associated risk of morbidities.

This study has several limitations. First, this study used manual segmentation, which is regarded as the gold standard. However, it may have suffered from significant interreader bias and is time-consuming. Although automatic segmentation is fast, accuracy and reproducibility should be considered. Second, due to the retrospective study design, potential selection bias might have been present. Third, the methodology of this study was mainly restricted to ML algorithms, and advanced deep learning (DL) methods can provide an end-to-end approach without complicated preprocessing steps. Thus, DL models should be investigated in future studies. Fourth, we did not thoroughly investigate the biological process behind each selected radiological and radiomic feature. Further experiments such as radiogenomics analysis may be required to solve this issue, which may further enhance our understanding of the disease. Fifth, we did not perform for predicting recurrence risk of meningioma due to the small number of patients with recurrent meningioma. Therefore, a larger number of recurrent patients in a prospective study may be necessary to further investigation.

In conclusion, this study has developed clinical-radiomic ML models to non-invasively predict the expression of Ki-67 and p53 in meningioma using mpMRI features and provides a novel non-invasive strategy for assessing cell proliferation.

## Data availability statement

The raw data supporting the conclusions of this article will be made available by the authors, without undue reservation.

## Ethics statement

The studies involving human participants were reviewed and approved by Chonnam National University Hospital. The patients/participants provided their written informed consent to participate in this study.

## Author contributions

CMM, YYL, SSS, and SKK designed the study; CMM and YYL performed the majority of experiments; CMM, YYL, DYK, WY, BHB, JHP, and SHH contributed to the analysis and interpretation of results; CMM and YYL wrote the first draft of the manuscript; SSS and SKK have approved the final manuscript and completed manuscript. Also, all authors agree with the content of the manuscript. All authors contributed to the article and approved the submitted version.
