# Supplementary material for: Preoperative prediction of Ki-67 and p53 status in meningioma using a multiparametric MRI-based clinical-radiomic model
Source: Front Oncol. 2023 May 23;13:1138069. doi: 10.3389/fonc.2023.1138069 (PMC10241997; doi:10.3389/fonc.2023.1138069)
Supplement: Supplementary file 1 [file Image_1.pdf]

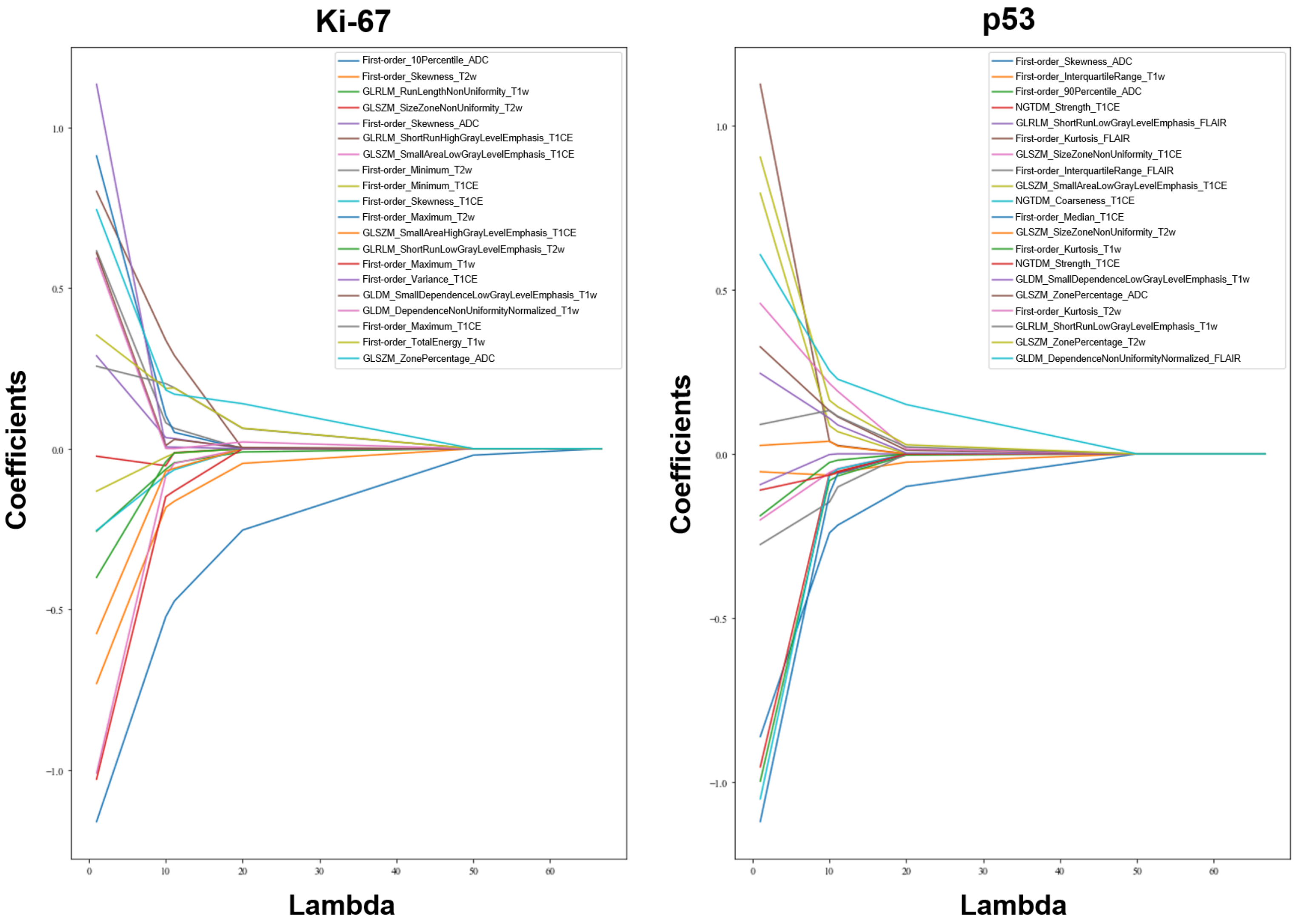

Supplementary Figure 1. The coefficients lambda to affect the final prediction in Ki-67 and p53, respectively.
